# Anwuligan inhibits the progression of non‐small cell lung cancer via let‐7c‐3p/PI3K/AKT/mTOR axis

**DOI:** 10.1002/cam4.5382

**Published:** 2022-11-22

**Authors:** Huikun Niu, Dexiang Wang, Tingting Wen, Han Liu, Jing Jie, Lei Song, Dan Li

**Affiliations:** ^1^ Department of Respiratory Medicine, Center for Pathogen Biology and Infectious Diseases, Key Laboratory of Organ Regeneration and Transplantation of the Ministry of Education The First Hospital of Jilin University Changchun China; ^2^ Department of Pulmonary and Critical Care Medicine Qilu Hospital, Cheeloo College of Medicine, Shandong University Jinan China

**Keywords:** anwuligan, let‐7c‐3p, PIK3CA

## Abstract

**Background:**

Anwuligan (ANW) isolated from nutmeg, also known as myristyl lignan, has attracted attention due to its therapeutic potential in diseases. However, its effect on lung cancer is still unclear.

**Methods:**

In this study, the cytotoxicity of ANW on non‐small cell lung cancer (NSCLC) cells was detected using a Cell Counting Kit‐8 (CCK‐8) assay. In vitro, clone formation, wound healing, and Transwell assays were used to investigate the migratory and invasive abilities of NSCLC cells after ANW treatment. The expression levels of the associated proteins were detected using Western blotting. A luciferase assay was used to validate the binding of let‐7c‐3p to the 3′‐untranslated region (UTR) of PIK3CA. In vivo, an A549 cell xenograft mouse model was used to verify the effect of ANW on tumor growth.

**Results:**

The results showed that ANW treatment (20 or 50 μM) had obvious cytotoxicity against A549 and H460 cells, suppressing cell proliferation and migration in vitro. In vivo, the growth of the implanted tumor was inhibited by ANW in a nude mouse model. The growth of NSCLC cells was also inhibited by let‐7c‐3p overexpression in vitro and in vivo. The inhibitory effect of ANW on the proliferation and metastasis of NSCLC cells was weakened by the downregulation of let‐7c‐3p, whereas it was enhanced by the overexpression of let‐7c‐3p; PIK3CA was the main target of let‐7c‐3p.

**Conclusions:**

In summary, ANW inhibits the growth and metastasis of NSCLC cells in vivo and in vitro by upregulating the expression of let‐7c‐3p, which can regulate the PI3K/AKT/mTOR signaling pathway. PIK3CA is the main target of let‐7c‐3p.

## Graphical abstract

Anwuligan inhibits the growth and metastasis of NSCLC cells by upregulating the expression of let‐7c‐3p, which can bind to the 3′‐untranslated region (UTR) of PIK3CA and downregulate the PI3K/AKT/mTOR signalling pathway.

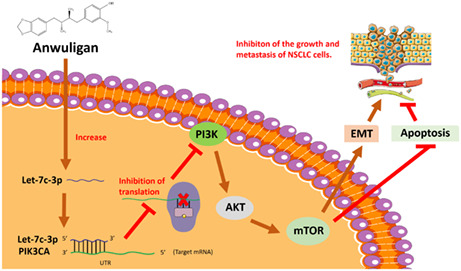

## 1 BACKGROUND

Lung cancer is one of the most common malignant tumors worldwide, contributing to 11.4% of newly diagnosed cancers and almost 18% of cancer‐related deaths.^1^ Non‐small cell lung cancer (NSCLC) is the main subtype of lung cancer, with 80%–85% of lung cancers being NSCLC. Although treatment strategies for this disease have advanced in recent years, the overall survival rate of patients with NSCLC has only poorly improved. Therefore, the development of new drugs with fewer side effects is necessary to treat NSCLC.

MicroRNAs (miRNAs) are short RNAs consisting of 19–25 nucleotides.^2^ They are known to regulate essential biological processes in plants and animals.^3^ MicroRNAs can regulate gene expression after transcription^2^ and influence many biological functions, such as cell survival, proliferation, apoptosis, and the growth and metastasis of tumors.^4^ Anti‐miRs targeting miRNAs have shown good prospects in preclinical development. For example, anti‐miRs targeting miR‐122 have been applied in phase II clinical trials to treat hepatitis, and mimics of miR‐34, a tumor suppressor miRNA, have been applied in phase I clinical trials for cancer treatment.^5^ Much research data has shown that miRNAs can suppress tumor growth, prevent cancer progression, and act as the biomarkers for the diagnosis and prognosis of lung cancer. Furthermore, miRNAs do not only regulate the metabolism of cancer cells but also mediate the resistance or sensitivity of cancer cells to chemotherapy and radiotherapy.^6^ However, clinical trials involving miRNAs targeting lung cancer have not shown promising results^7^; therefore, further research is needed to prove whether patients with lung cancer could benefit from miRNA‐targeted therapy.

Phosphatidylinositol 3‐kinase (PI3K), and its downstream effector, serine/threonine protein kinase B (PKB, also known as AKT), comprise the PI3K‐AKT–mTOR signaling pathway. Mammalian target of rapamycin (mTOR) is a serine/threonine protein kinase belonging to the PI3K‐related kinase protein family. It acts as a regulator of cell growth and proliferation and receives nutrition‐related signals.^8^ PI3K/AKT/mTOR signal transduction is one of the key pathways driving the malignant process and drug resistance in patients with solid tumors. The PI3K/AKT/mTOR signaling pathway is important in aspects of cell survival and growth, resulting in various pathological and physiological conditions.^9^ In NSCLC, the PI3K/AKT/mTOR pathway is closely associated with tumorigenesis and disease progression. Several specific PI3K, AKT, and mTOR inhibitors are currently under clinical development, although no promising results have yet been observed.^10^

Natural compounds have many complex chemical structures, some of which have great potential for cancer treatment. Many studies have indicated that phytochemicals exert anti‐cancer effects by influencing specific target genes and signaling pathways related to tumorigenesis and cancer progression.^11^ Nutmeg, a tropical evergreen herb, is native to Indonesia but can also be found in Iran, India, South America, and the West Indies. It also exhibits antifungal and antibacterial activities.^12^ Anwuligan (ANW), a compound isolated from nutmeg, also known as myristyl lignan, has warranted attention because of its therapeutic potential in many diseases. It has been proven to have antioxidant, antimicrobial, anti‐inflammatory, anti‐caries, and hepatoprotective properties.^13^ ANW has been shown to induce the apoptosis of human promyelocytic leukemia cells (HL‐60) through the activation of caspase‐3, demonstrating its anti‐cancer activity. In addition, ANW attenuated cisplatin‐induced hepatotoxicity.^14^ Despite these evidence of the anti‐cancer properties of ANW, its therapeutic effect in lung cancer remains unclear. This study demonstrated inhibitory effect of ANW treatment in lung cancer in vivo and in vitro and explored the underlying mechanisms to clarify its importance in NSCLC treatment.

## 2 MATERIALS AND METHODS

### 2.1 Cell culture and treatment

Two human non‐small‐cell lung cancer cell lines (A549 and H460), a human bronchiolar epithelial cell line (BEAS‐2B), and a human embryonic lung fibroblast line (MRC‐5) were provided by the Chinese Academy of Sciences. All cells were cultured in Dullbecco's Modified Eagle Medium (DMEM, Gibco) containing 1% penicillin–streptomycin (Hyclone) and 10% fetal bovine serum (Gibco) in a cell incubator with 5% CO_2_ and 37°C.

The effects of anwuligan (Herbpurify, China) on NSCLC in vitro were explored by treating NSCLC cells with a vehicle and different doses of ANW (5, 20, 50, or 100 μM). To determine whether ANW could enhance the effect of cisplatin on NSCLC cells, different concentrations of Anwuligan (0, 5, 20, 50 μM) and cisplatin (10 μg/mL) were added to the NSCLC cells.

### 2.2 Cell transfection and transduction

Approximately 1 × 10^5^ A549 and H460 cells were seeded per well of six‐well plates and cultured for 24 h before transfection and transduction. The let‐7c‐3p mimic and its scrambled control and mimic NC, as well as the let‐7c‐3p inhibitor (2′‐O‐methyl‐modified) and NC inhibitor, were provided by RiboBio Co., Ltd., and the cells were divided into four groups (negative control group, ANW group, ANW + let‐7c‐3p inhibitor NC group, and ANW + let‐7c‐3p inhibitor group). In addition, pCDH‐PIK3CA lentiviral particles packaged with the recombinant vector and pCDH lentiviral particles packaged with the empty vector were transduced into the cells. Lipofectamine® 2000 (Invitrogen) was used for transfection.

### 2.3 CCK‐8 assay

Before the corresponding treatments, approximately 5 × 10^3^ MRC‐5, BEAS‐2B, A549, and H460 cells were seeded per well of 96‐well plates and cultured at 37°C for 24 h. After treatment, the cells were incubated for 0, 24, 48, or 72 h, and cell proliferation was measured using a CCK‐8 kit (MCE, USA). Briefly, 10 μl CCK‐8 solution was added to each well, and the plates were incubated for 2 h. The absorbance of each well was measured at 450 nm with a microplate reader (*n* = 3 per group; results are presented as the mean of 3 independent experiments).

### 2.4 EdU assay

A549 and H460 cells were seeded in 96‐well plates at a density of 5 × 10^3^ cells per well and cultured at 37°C with 5% CO_2_ for 24 h before being subjected to the respective treatments. After 24 h, 100 μl of complete culture medium containing 10 μM EdU was added to each well, and the cells were incubated for another 2 h. After incubation, the cells were fixed with 4% paraformaldehyde for 30 min and permeabilized via incubation with 0.5% Triton X‐100 for 20 min at room temperature. Hoechst 33342 was then used to counterstain the nuclei. An inverted fluorescence microscope (Olympus) was used to obtain images.

### 2.5 Clone formation assay

NSCLC cells were inoculated in six‐well plates (200 cells per well) and incubated at 37°C. After 24 h, the cells were subjected to the corresponding treatments and incubated for 2 weeks. The cells were then fixed with 4% paraformaldehyde for 20 min and stained with 0.1% crystal violet for 15 min at room temperature. The number of colonies was quantified using ImageJ software.

### 2.6 Cell cycle detection

Before treatment, A549 and H460 cells were inoculated in six‐well plates and cultured for 24 h. After incubation, the cells were digested with trypsin and collected in centrifuge tubes. Then, 75% ethanol was added to fix the cells overnight. The next day, the cells were stained with propidium iodide (PI) for 30 min at 37°C and analyzed using flow cytometry.

### 2.7 Wound healing assay

A549 and H460 cells in the logarithmic phase were seeded in six‐well plates and incubated at 37°C until the cell monolayer reached approximately 90% confluence. After treatment, the cell monolayers were subject to uniform, straight scratches using pipette tips, and washed with phosphate‐buffered saline (PBS) to remove cell debris. Next, the cells were incubated with 2 ml complete culture medium for an additional 24 h. Images were obtained using an inverted microscope at 0 and 24 h. ImageJ software was used to assess the rate of wound healing.

### 2.8 Transwell assay

Here, 24‐well Transwell plates containing Matrigel were used to assess the migration and invasion abilities of NSCLC cells. After digestion and collection, A549 and H460 cells were resuspended in serum‐free medium and adjusted to the desired cell density (2 × 10^5^ cell/mL). After treatment, 100 μl of the cell suspension was added to the upper chamber of each Transwell. The bottom chamber was filled with a medium supplemented with 10% fetal bovine serum, and the cells were incubated for 48 h. After incubation, the cells in the upper chamber were wiped off with a cotton swab, while the cells in the lower chamber were fixed with 10% pre‐chilled paraformaldehyde for 30 min. The cells were then stained with 0.1% crystal violet. Finally, images were obtained using an inverted microscope, and the number of stained cells in five random fields was counted.

### 2.9 Detection of cell apoptosis

A549 and H460 cells were seeded in six‐well plates at a density of 2 × 10^5^ cell/mL and cultured at 37°C. After 24 h, the cells were subjected to the prescribed treatments and incubated for 24 h. Images were obtained using an inverted microscope to assess cell morphology. The medium containing the floating apoptotic and anchorage‐dependent cells was collected in centrifuge tubes. After centrifugation, the desired cell precipitate was obtained. Subsequently, the cells were resuspended in pre‐cooled PBS and centrifuged. An Annexin V‐FITC Apoptosis Kit (Invitrogen) was used to detect cell apoptosis. The cell pellet was resuspended in 1× binding buffer, and the cell density was adjusted to a density of 3 × 10^5^ cells/mL. Next, 100 μl of the cell suspension was removed and added to clean Eppendorf tubes. In each tube, 5 μl of annexin V‐FITC was used to stain the cells for 15 min, then 5 μl of PI was used to stain these cells for 5 min. Subsequently, the samples were resuspended with 200 μl of 1× binding buffer and cell apoptosis was analyzed via flow cytometry.

### 2.10 Western blot

The total protein concentration derived from NSCLC cells was tested using a bicinchoninic acid protein assay kit (Tian Gen). In brief, 30 μg total protein was added to the relevant sample wells, electrophoretically separated using a 10% sodium dodecyl sulfate‐polyacrylamide gel, and transferred to a polyvinylidene fluoride membrane (Bio‐Rad, USA) through a semi‐dry electrophoretic transfer. The membrane was immersed in the 5% melted skim milk powder, and then the membranes were probed with the primary antibodies on a shaker at 4°C overnight. The next day, the membranes were washed three times with phosphate‐buffered saline containing Tween‐20 and incubated with the corresponding secondary antibodies for 2 h at room temperature. Two minutes after covering the film with a luminescent developer, a gel‐imaging system was used to visualize the protein bands.

### 2.11 Prediction of the target genes of let‐7c‐3p

Target genes of let‐7c‐3p were predicted using PicTar (https://pictar.mdc‐berlin.de/), miRBase (http://www.mirbase.org/), and TargetScanHuman (version 7.2;http://www.targetscan.org/vert_72/).

### 2.12 qRT‐PCR analysis

Before qRT‐PCR, NSCLC cells were treated with different concentrations of anwuligan and then incubated for 24 h. Total RNA was extracted using TRIzol reagent (Invitrogen), and reverse transcribed into cDNA using a PrimeScript II 1st Strand cDNA Synthesis kit (Takara). PowerUp SYBR Green Master Mix (Applied Biosystems) was used for qRT‐PCR. U6 and GAPDH were used as internal controls to measure the let‐7c‐3p and PIK3CA expression levels, respectively. The 2^−∆∆Ct^ method was used to quantify relative expression levels.

### 2.13 Deep sequencing

Deep sequencing was performed to investigate the effects of anwuligan on the miRNA expression spectrum of NSCLC cells. The NSCLC cells were divided into experimental and control groups. The cells were treated with anwuligan (50 μM) for 12 h, the total RNA was isolated, and the total RNA was sent for deep sequencing by the Capital Bio Technology Company.

### 2.14 Luciferase reporter assay

The 3′‐UTR region of PIK3CA containing the wild‐type (WT) and mutant (MT) let‐7c‐3p binding sites was cloned using PCR and inserted into the pmiR‐RB‐REPORT vector (Ambion). The resulting pmiR‐RB‐REPORT‐PIK3CA‐mut/WT/si vectors were then co‐transfected with the let‐7c‐3p mimic, scrambled control, or pRL‐TK into HEK293T cells, which were then incubated for 24 h. A Dual Luciferase Assay Kit (Promega) was used to detect luciferase activity.

### 2.15 Establishment of a nude mouse xenograft tumor model

BALB/c nude mice aged 4 weeks (12 males, 12 females) were provided by the Jilin University Animal Center and raised in laboratory animal barrier system facilities. All animal experiments were approved by the Animal Protection and Utilisation Committee of the First Hospital of the Jilin University. A549 cells at a concentration of 5 × 10^5^ cells/ml diluted in PBS were subcutaneously injected into the BALB/c nude mice (1 × 10^6^ cells per mouse). Seven days after injection, the mice were randomly divided into four groups (*n* = 6 mice in each group). Either DMSO or anwuligan was injected into nude mice intraperitoneally at a dose of 20 mg/kg. In addition, normal saline, let‐7c‐3p antagomir, and antagomir NC (5000 nM) were injected intratumorally into the implanted tumor at multiple points every other day for a total of six times throughout the experimental period. Three days later, the tumor volumes were measured using a Vernier caliper and calculated according to the following formula: Tumor volume (mm^3^) = maximal length (mm) × [perpendicular width (mm)]^2^/2. On the 28th day after injection, the tumors were excised and photographed.

### 2.16 H&E staining

After sacrifice, the hearts, livers, and kidneys of the nude mice were collected and sectioned to three micron. The sections were stained with hematoxylin and eosin (H&E), and images were captured using a microscope.

### 2.17 Statistical analysis

Data were analyzed using GraphPad Prism software (version 6.02; GraphPad). The results are expressed as the mean ± standard deviation. Comparisons between two groups were performed using the *t*‐test, and analysis of variance was performed when comparing between more than two groups. *p* < 0.05 (*) and *p* < 0.01 (**) were considered statistically significant.

## 3 RESULTS

### 3.1 Anwuligan inhibits the growth and metastasis of NSCLC cells in vitro

**FIGURE 1:**
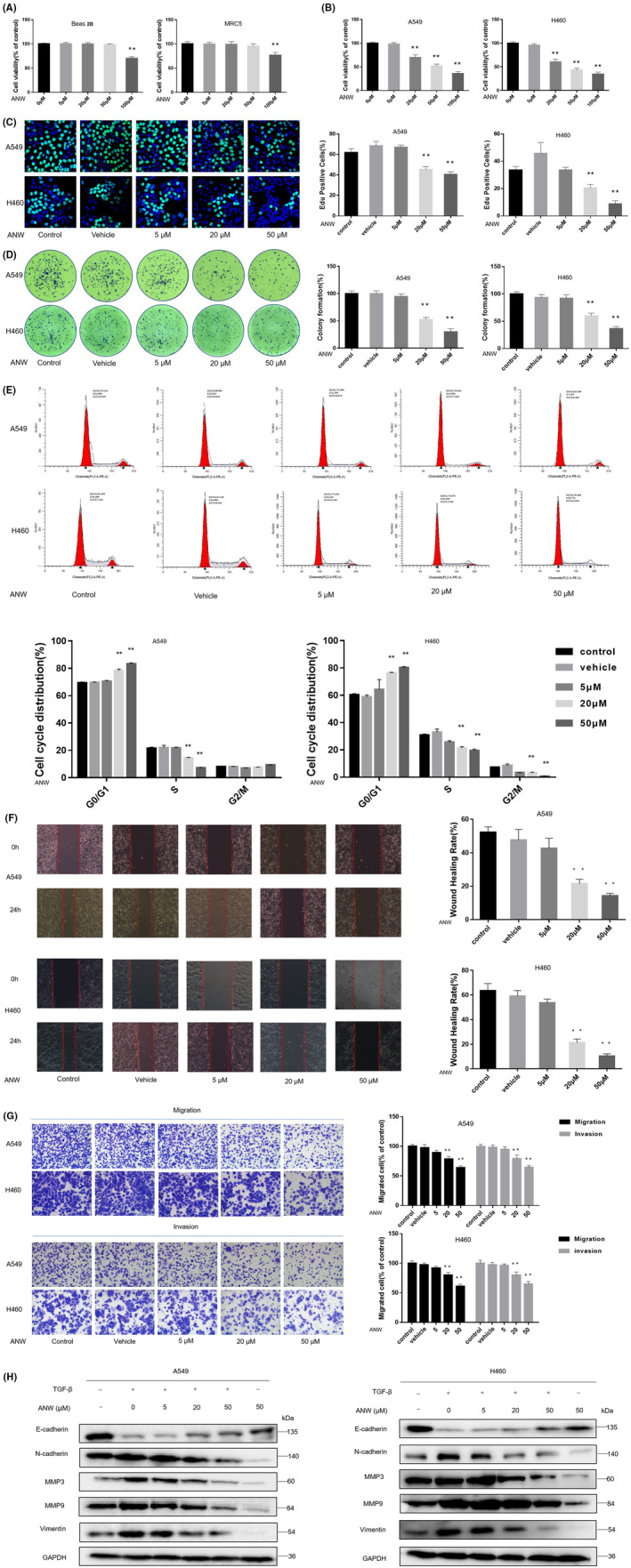
Anwuligan inhibits the proliferation and viability of non‐small cell lung cancer (NSCLC) cells in vitro. (A) After treatment with different concentrations of ANW for 48 h, the survival rate of normal lung cell lines (Beas 2B and MRC‐5) was detected using a CCK‐8 assay (*n* = 3). (B) After treatment with different concentrations of ANW for 48 h, the survival rate of NSCLC cells (A549 and H460) was detected at 450 nm using a CCK‐8 assay (*n* = 3). (C) Detection of EdU fluorescence in NSCLC cells (A549 and H460) after treatment with different concentrations of ANW (*n* = 3). (D) Detection of the clone formation rate of NSCLC cells (A549 and H460) after treatment with different concentrations of ANW (*n* = 3). (E) Cell cycle distribution of NSCLC cells after treatment with different concentrations of ANW was analyzed via flow cytometry (*n* = 3). (F) A wound healing assay was used to detect the effects of different concentrations of ANW on the migration of NSCLC cells (A549 and H460) (*n* = 3). (G) The Transwell assay was used to detect the effects of different concentrations of ANW on the migration and invasion ability of the NSCLC cells (A549 and H460) (*n* = 3). (H) The effects of different concentrations of ANW on the EMT‐related proteins induced by TGF‐β were detected using a Western blot (*n* = 3).

In this study, 48 h after treatment with 20 μM, 50 μM, and 100 μM ANW, the viability of A549 and H460 cells decreased markedly (Figure 1B). However, 48 h after treatment, the viability of Beas 2 B and MRC5 cells did not change appreciably until the ANW concentration reached 100 μM (Figure 1A). The EdU assay revealed that when 20 μM and 50 μM ANW were used to treat A549 and H460 cells, the proportion of fluorescent cells decreased notably (Figure 1C). In the clone formation assay, when the concentration of anwuligan reached 20 μM, the number of clones was remarkably lower than that in the control group. When the concentration of anwuligan was 50 μM, the reduction was more pronounced (Figure 1D).

Moreover, the cell cycle distribution was analyzed via flow cytometry (PI single staining). The results showed that the percentage of A549 and H460 cells treated with 20 μM and 50 μM ANW in the G0/G1 phase increased compared with the control group, suggesting that anwuligan arrested NSCLC cell growth at the G0/G1 phase (Figure 1E). The results of the wound healing assay revealed that the cell migration rate of A549 and H460 cells treated with 20 μM and 50 μM ANW were obviously lower than that of the control group (Figure 1F). Transwell assays revealed that the number of A549 and H460 cells that passed through the filter and Matrigel matrix was notably less than that in the control group (Figure 1G) after treatment with 20 μM and 50 μM ANW.

After the cells were treated with different concentrations of anwuligan and TGF‐β (transforming growth factor‐β; 5 ng/mL), a Western blot assay was conducted to detect the expression of epithelial‐mesenchymal transition (EMT)‐related marker proteins. The results revealed that treatment with 20 μM and 50 μM of anwuligan visibly inhibited TGF‐β‐mediated EMT, reducing the protein expression levels of N‐cadherin, MMP3, MMP9, and vimentin compared to untreated cells. Meanwhile, the levels of E‐cadherin in A549 and H460 cells treated with TGF‐β and anwuligan at doses of 20 μM and 50 μM were obviously higher than in cells treated with TGF‐β alone (Figure 1H). The results of above tests were dose‐dependent. These results revealed that ANW could inhibit the invasion and metastasis of NSCLC cells in a dose‐dependent manner, proving that anwuligan plays a role in inhibiting the growth and metastasis of NSCLC cells.

### 3.2 Anwuligan enhances the anti‐tumor effect of cisplatin on NSCLC cells

**FIGURE 2:**
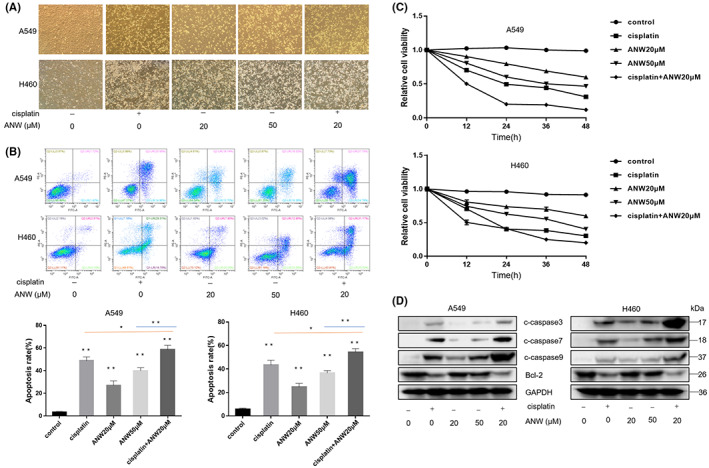
Anwuligan enhances the anti‐tumor effect of cisplatin on NSCLC cells. (A) The morphological changes of NSCLC cells treated with cisplatin and ANW for 24 hours were observed under a light microscope (*n* = 3). (B) Annexin V‐FITC/PI staining and flow cytometry were used to detect the apoptosis rate of NSCLC cells treated with cisplatin and ANW (*n* = 3). (C) The CCK‐8 assay was used to detect the viability of NSCLC cells treated with cisplatin and ANW (*n* = 3). (D)The expression of the pro‐apoptosis proteins caspase 3/7/9 and the anti‐apoptotic protein Bcl‐2 in NSCLC cells treated with ANW and cisplatin were detected using a Western blot assay (*n* = 3).

The morphology of A549 and H460 cells treated with cisplatin and anwuligan was observed under a light microscope. The results showed that the combination of 20 μM anwuligan and cisplatin (10 μg/mL) increased cell atrophy and decreased cell adhesion to a greater extent compared with treatment using cisplatin or anwuligan alone (Figure 2A). Annexin V‐FITC/PI staining and flow cytometry were also used to analyze the apoptosis rate of the NSCLC cells (A549 and H460) treated with cisplatin (10 μg/mL) and ANW. The results indicated that anwuligan significantly increased the proportion of apoptotic cells in a dose‐dependent manner. In addition, the proportion of apoptotic cells was the highest in the cells treated with 20 μM anwuligan along with cisplatin (10 μg/mL) (Figure 2B). The results of the CCK‐8 assay also showed that the combination of anwuligan and cisplatin (10 μg/mL) had a stronger effect on reducing cell viability (Figure 2C). The results of the Western blot assay also revealed that ANW increased the expression of apoptosis‐related protein caspases 3/7/9 and decreased the levels of the anti‐apoptotic protein B‐cell lymphoma‐2 (Bcl‐2), especially when combined with cisplatin (Figure 2D). These results suggest that anwuligan treatment enhanced the cisplatin‐induced apoptosis of NSCLC cells.

### 3.3 Anwuligan upregulates let‐7c‐3p expression in NSCLC cells

**FIGURE 3:**
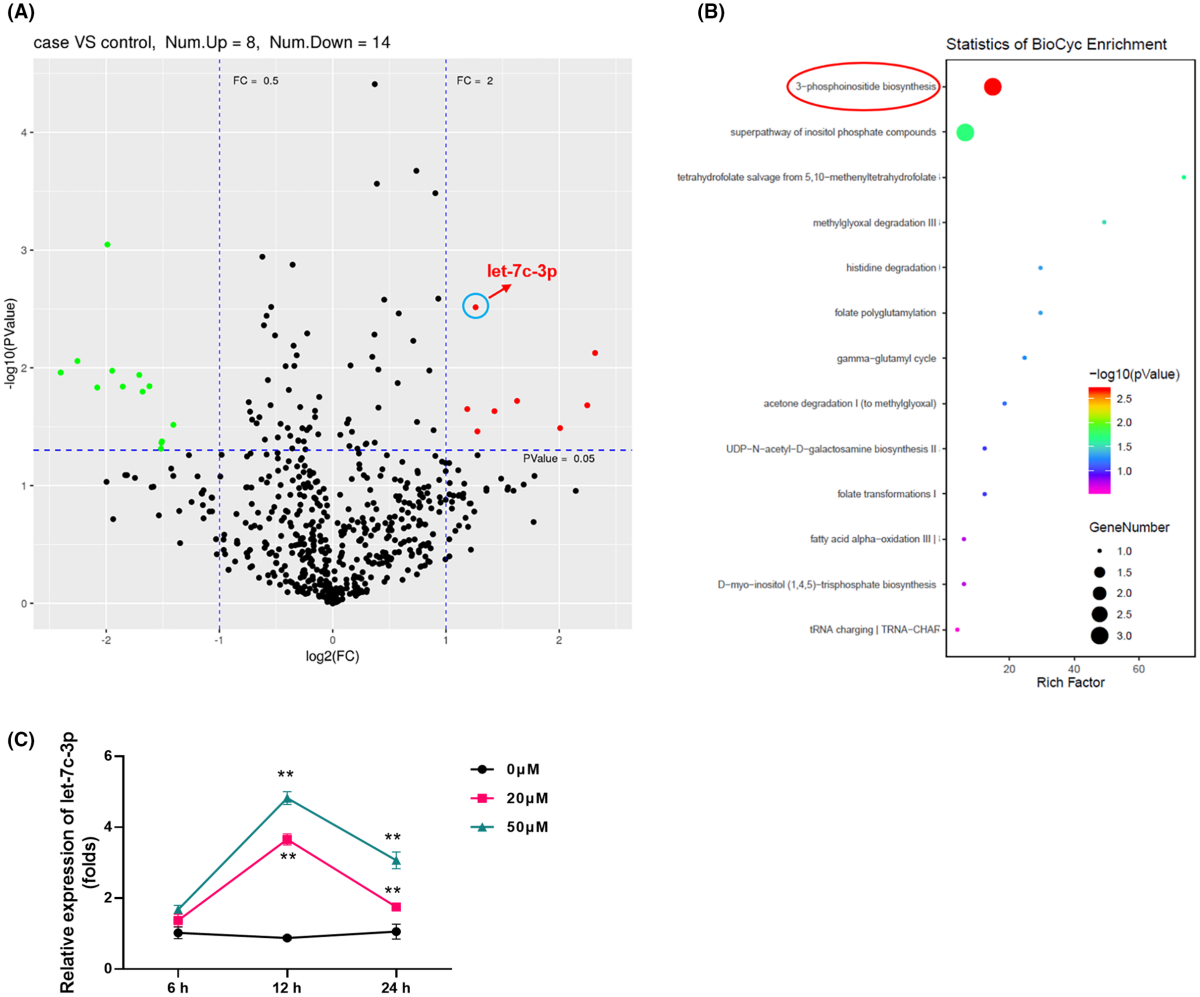
Anwuligan upregulates microRNA let‐7c‐3p expression in NSCLC cells. (A) Scatter plot of microRNA expression changes in A549 cells treated with ANW for 24 h as detected via deep sequencing. (B) Bioinformatics analysis and prediction of the function of let‐7c‐3p. (C) The expression of let‐7c‐3p in A549 cells stimulated with different concentrations of ANW (0, 20, or 50 μM) was assessed using qRT‐PCR.

The expression of microRNAs in A549 cells with or without treatment with anwuligan was compared using deep sequencing. The results indicated that after 24 h of treatment with anwuligan, the levels of 683 miRNAs in A549 cells changed. Out of these, the expression of 22 miRNAs changed more significantly than others (*p* < 0.05): Eight were upregulated and 14 were downregulated (Figure 3A). The specific changes of these 22 miRNAs are listed in Table 1. Among these miRNAs, the level of hsa‐let‐7c‐3p was one of the most noticeable. After verification via qPCR and pre‐experiment (proliferation and migration experiments), only let‐7c‐3p was found to play a significant role in NSCLC and was affected by pharmaceutical intervention. Thus, let‐7c‐3p was selected as the primary target in this study. Bioinformatics analysis showed that the change in hsa‐let‐7c‐3p levels was mainly associated with the biosynthesis of inositol 3‐phosphate (Figure 3B). To further confirm that ANW could play a role in increasing the expression of let‐7c‐3p in NSCLC cells, the expression of let‐7c‐3p was detected using qRT‐PCR after treatment at different ANW concentrations at different time points. The results indicated that the expression of let‐7c‐3p began to increase after 6 h of stimulation with anwuligan and that it peaked at 12 h. Moreover, let‐7c‐3p expression was increased by ANW treatment in a dose‐dependent manner (Figure 3C). These data indicate that anwuligan upregulates let‐7c‐3p expression in NSCLC cells.

**TABLE 1:** List of microRNAs with fold‐changes >2 after drug treatment

名称	log2FC	*p* Value	FC
UP			
hsa‐let‐7c‐3p	1.261174001	0.003058992	2.396907111
hsa‐miR‐181b‐2‐3p	1.187592906	0.022398949	2.277723945
hsa‐miR‐214‐3p	1.427874176	0.02333952	2.690499756
hsa‐miR‐3138	2.246871788	0.02080711	4.746525349
hsa‐miR‐337‐3p	1.27706704	0.034661081	2.423457934
hsa‐miR‐376a‐2‐5p	2.316003569	0.007473635	4.979509262
hsa‐miR‐627‐5p	1.627167362	0.019118596	3.089058862
hsa‐miR‐6516‐3p	2.007125304	0.032494875	4.019804402
DOWN			
hsa‐miR‐155‐3p	−1.51268059	0.04327436	0.350459445
hsa‐miR‐185‐3p	−1.406888448	0.030487024	0.377124178
hsa‐miR‐205‐5p	−1.516377485	0.048623053	0.349562545
hsa‐miR‐20a‐3p	−1.618637064	0.014335344	0.325642959
hsa‐miR‐3184‐3p	−2.079221953	0.014688993	0.236641999
hsa‐miR‐320a‐5p	−1.946504317	0.010590038	0.259444109
hsa‐miR‐374c‐5p	−1.507981846	0.042149115	0.351602724
hsa‐miR‐4448	−1.853581327	0.014437963	0.276704627
hsa‐miR‐450b‐5p	−1.988843781	0.000897069	0.251940719
hsa‐miR‐548ad‐5p	−1.678686692	0.015928661	0.31236686
hsa‐miR‐548ae‐5p	−2.253683813	0.008748639	0.209687997
hsa‐miR‐548ay‐5p	−2.401589008	0.010963183	0.189256006
hsa‐miR‐548d‐5p	−2.401589008	0.010963183	0.189256006
hsa‐miR‐6894‐5p	−1.7077901	0.011492572	0.306128634

### 3.4 Anwuligan inhibits the PI3K/AKT/mTOR signaling pathway in NSCLC cells

**FIGURE 4:**
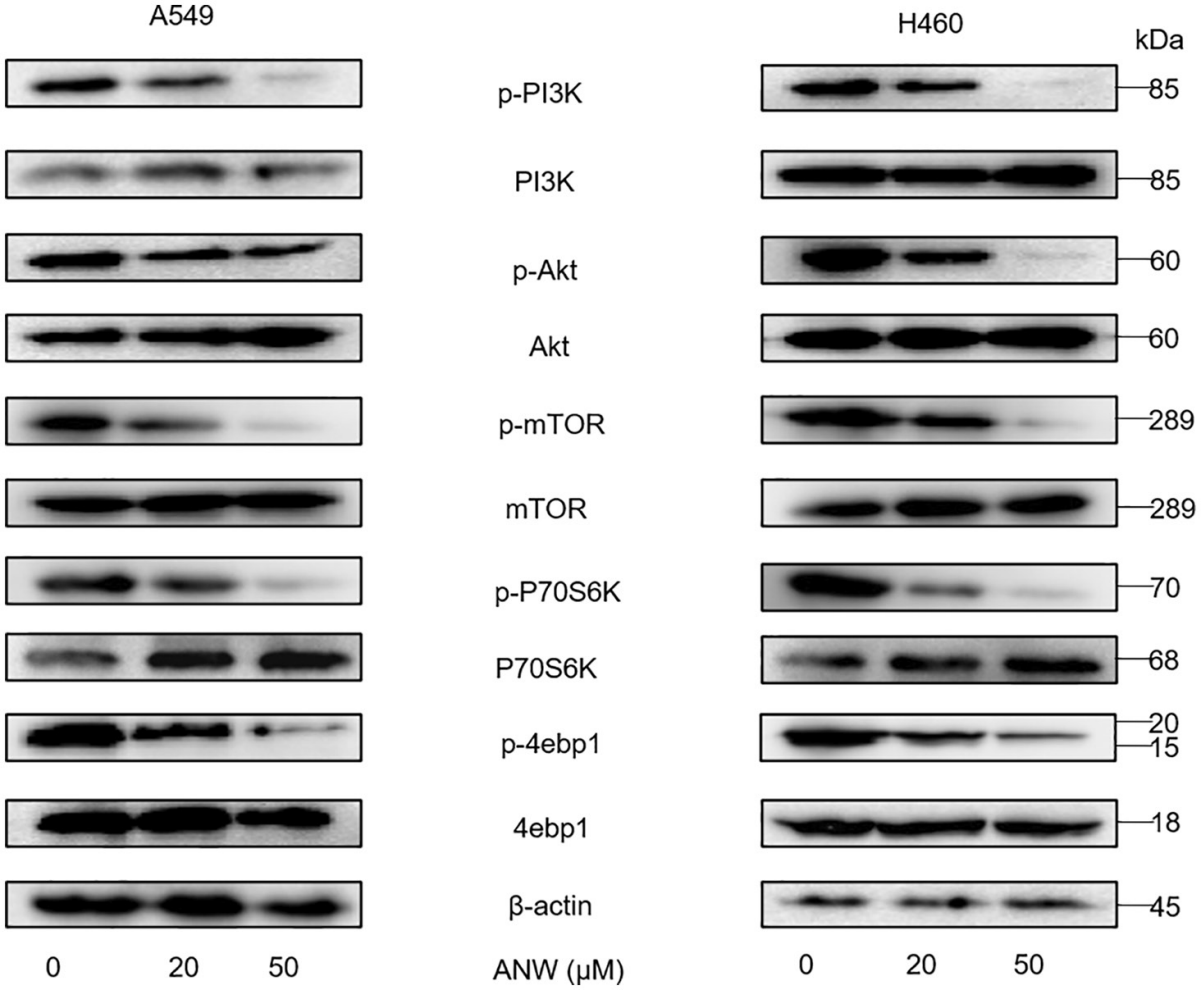
Anwuligan inhibits the PI3K/AKT/mTOR signaling pathway in NSCLC cells. Western blot of proteins related to the PI3K/AKT/mTOR signalling pathway in A549 and H460 cells treated with different concentrations of ANW (0, 20, and 50 μM) for 24 h (*n* = 3).

The western blot results revealed that the phosphorylation levels of PI3K, AKT, mTOR, p70S6K, and 4 E‐BP1 in A549 and H460 cells treated with ANW were significantly reduced compared with untreated cells in a dose‐dependent manner (Figure 4). This result indicates that ANW treatment can play a role in inhibiting the PI3K/AKT/mTOR signaling pathway in NSCLC cells.

### 3.5 let‐7c‐3p inhibits the PI3K/AKT/mTOR pathway by directly targeting PIK3CA

**FIGURE 5:**
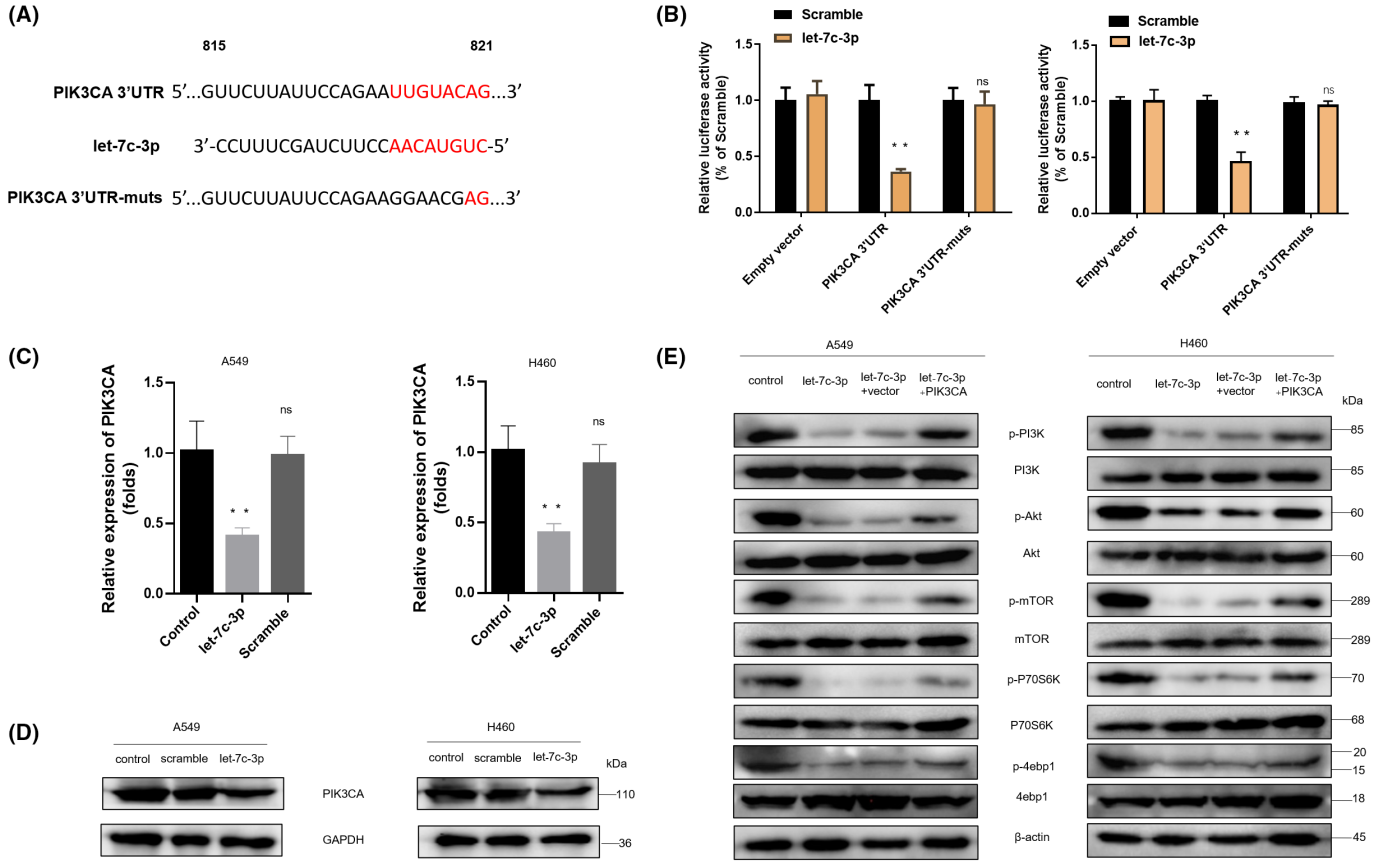
miRNA let‐7c‐3p inhibits the PI3K/AKT/mTOR pathway by directly targeting PIK3CA. (A) MicroRNA.org, Targetscan, and miRbase were used to analyze and predict the possible target genes and binding sites of let‐7c‐3p, the PIK3CA 3′‐UTR sequence, and the corresponding let‐7c‐3p 5′‐end, and the PIK3CA 3′‐UTR mutation sequence. (B) A luciferase gene reporter assay was used to detect the effect of let‐7c‐3p on the luciferase activity of the PIK3CA 3′‐UTR sequence. (C) The effect of let‐7c‐3p on PIK3CA expression in A549 and H460 cells was assessed using qRT‐PCR. (D) Western blot was used to detect the effect of let‐7c‐3p overexpression on PIK3CA expression in A549 and H460 cells (*n* = 3). (E) Western blot was used to assess the influence of let‐7c‐3p and PIK3CA overexpression on the expression of proteins related to the PI3K/AKT/mTOR signaling pathway in A549 and H460 cells (*n* = 3).

MicroRNA.org, TargetScan, and miRbase software were used to predict the possible target genes of let‐7c‐3p. Our analyses showed that 8‐nt‐long fragments (nt 815–821) matching let‐7c‐3p were found at the 3′‐UTR of PIK3CA (Figure 5A). The luciferase gene reporter assay results in HEK293T and A549 cells showed that let‐7c‐3p significantly decreased the luminescence of the PIK3CA‐3′UTR‐wt group compared with the PIK3CA‐3′UTR‐mut and empty vector groups (Figure 5B). After the let‐7c‐3p mimic and scrambled control were transfected into A549 and H460 cells, the expression level of PIK3CA in the cells was assessed using qRT‐PCR. The results revealed that, compared with the control group, the level of PIK3CA in the let‐7c‐3p group was significantly downregulated, but there were not significantly different in the level of PIK3CA between the control group and the scramble group (Figure 5C). Western blot analysis revealed that the expression of PIK3CA in the let‐7c‐3p group was significantly lower in the control and scrambled control groups (Figure 5D). Next, a Western blot assay was also conducted to determine whether let‐7c‐3p could regulate the expression of proteins associated with the PI3K/AKT/mTOR signaling pathway by regulating the expression of PIK3CA. The results showed that in A549 and H460 cells, let‐7c‐3p reduced the phosphorylation levels of PI3K, AKT, mTOR, P70S6K, and 4ebp1, which could be suppressed by the overexpression of PIK3CA (Figure 5E). In summary, let‐7c‐3p inhibits the PI3K/AKT/mTOR pathway by directly targeting PIK3CA in NSCLC cells.

### 3.6 let‐7c‐3p inhibits the proliferation and metastasis of NSCLC cells

**FIGURE 6:**
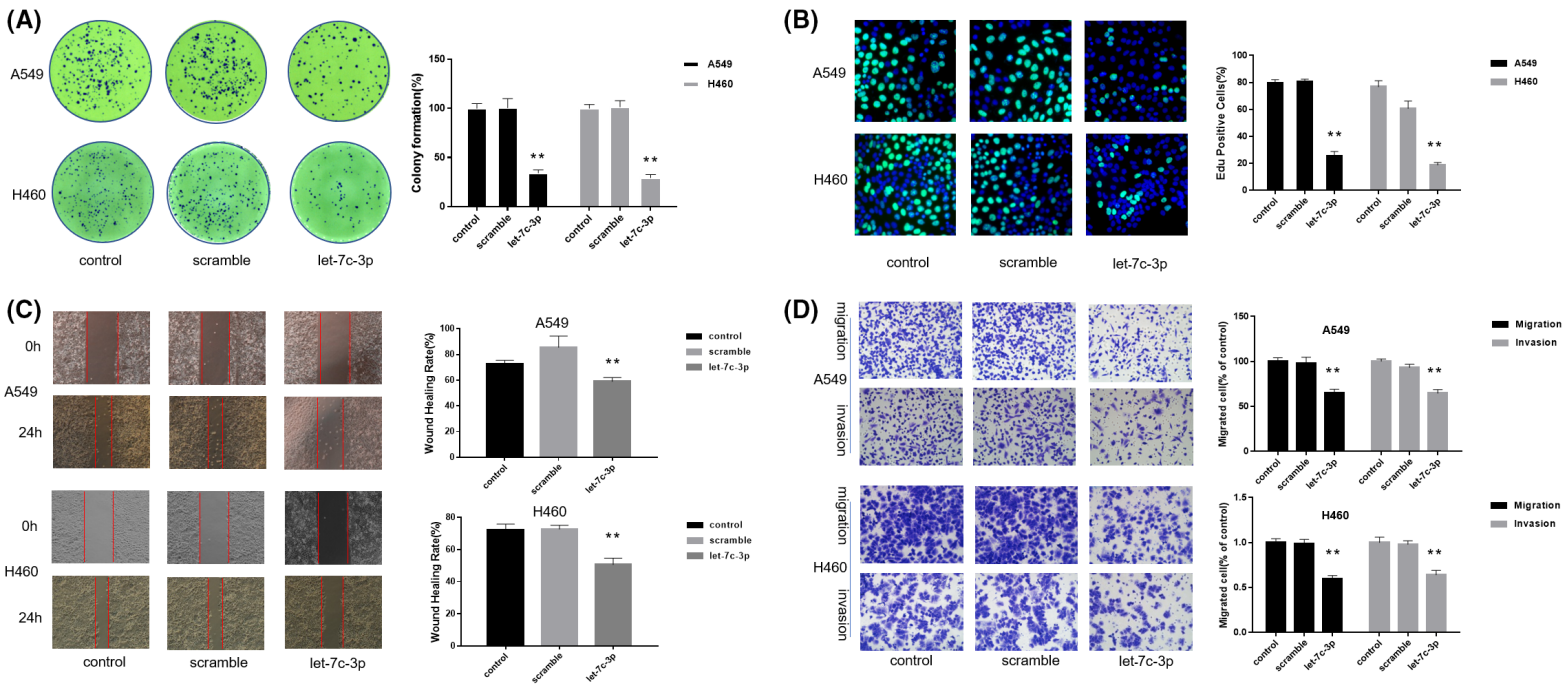
miRNA let‐7c‐3p inhibits the proliferation and metastasis of NSCLC cells. Here, let‐7c‐3p and a scrambled control were transfected into A549 and H460 cells, and a blank control group was added for the experiment. (A) To detect the effect of let‐7c‐3p overexpression on the cell clone formation ability of NSCLC cells (*n* = 3). (B) EdU staining was used to detect if the overexpression of let‐7c‐3p affects the proliferation of NSCLC cells (*n* = 3). (C) The wound healing assay was used to detect the effect of let‐7c‐3p overexpression on the migration ability of NSCLC cells (*n* = 3). (D) The Transwell assay was used to detect whether the overexpression of let‐7c‐3p affects the migration and invasion ability of NSCLC cells (*n* = 3).

As mentioned earlier, let‐7c‐3p inhibited the PI3K/AKT/mTOR pathway by targeting PIK3CA in A549 and H460 cells. To verify whether let‐7c‐3p affects the growth of NSCLC cells, let‐7c‐3p and a scrambled control were transfected into A549 and H460 cells. The results of the clone formation assay showed that the number of A549 and H460 cell clones in the scrambled control and control groups was significantly higher than that in the let‐7c‐3p group, and there was no obvious difference between the scrambled control and control groups (Figure 6A). The results of the EdU assay showed that the proportion of fluorescent cells in the let‐7c‐3p group was lower than that in the control and scrambled control groups (Figure 6B). The results of the wound healing assay showed that let‐7c‐3p significantly decreased the wound healing rate of NSCLC cells compared with the control and scrambled control groups (Figure 6C). In addition, the results of the Transwell assay indicated that the number of migrating and invading cells in the let‐7c‐3p group was less than that in the control and scrambled control groups (Figure 6D). These data suggest that let‐7c‐3p overexpression inhibits the proliferation and metastasis of NSCLC cells.

### 3.7 Inhibiting let‐7c‐3p counteracts the anti‐NSCLC cell effect of anwuligan

**FIGURE 7:**
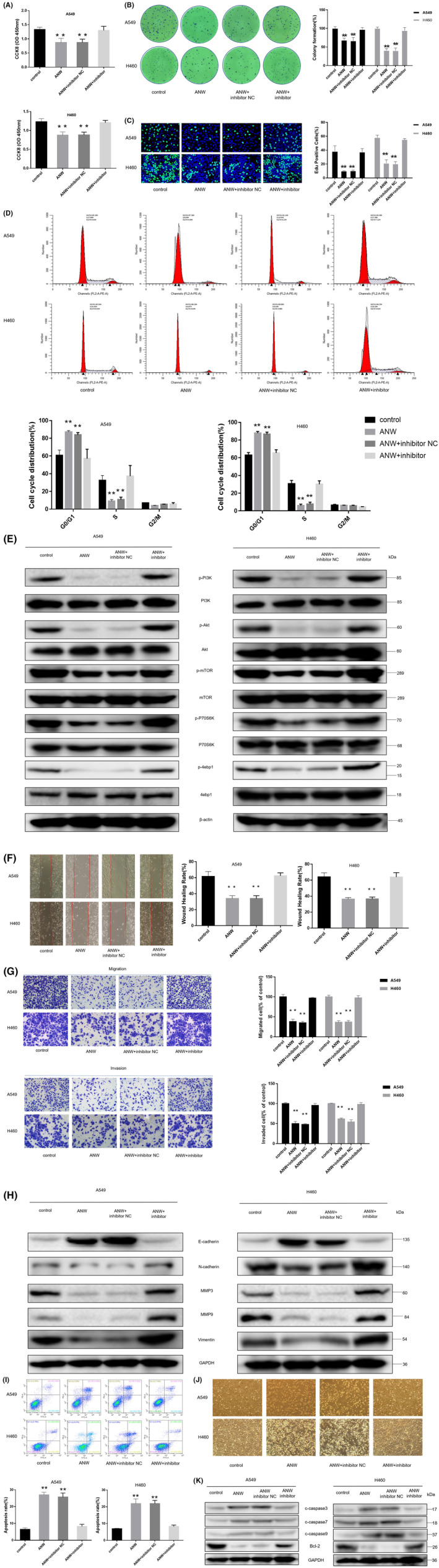
Inhibition of let‐7c‐3p counteracts the anti‐NSCLC cell effect of anwuligan. (A) The CCK‐8 assay was used to detect the effect of let‐7c‐3p on the viability of NSCLC cells treated with anwuligan (*n* = 3). (B) Detection of the clone formation rate of NSCLC cells (A549 and H460) in the control, ANW, ANW + let‐7c‐3p inhibitor, and ANW + let‐7c‐3p inhibitor NC groups (*n* = 3). (C) Detection of EdU fluorescence in NSCLC cells of the ANW, ANW + let‐7c‐3p inhibitor, and ANW + let‐7c‐3p inhibitor NC groups. (D) Flow cytometry was performed to detect the effect of let‐7c‐3p inhibition on the cell cycle distribution of NSCLC cells treated with anwuligan (*n* = 3). (E) A Western blot assay was used to detect the expression levels of PI3K/AKT/mTOR signalling pathway‐related proteins in the four groups (*n* = 3). (F) The wound healing assay was used to detect the effect of let‐7c‐3p inhibition on the migration of ANW‐treated NSCLC cells (*n* = 3). (G) A Transwell assay was used to detect the effect of let‐7c‐3p inhibition on the migration and invasion ability of ANW‐treated NSCLC cells (*n* = 3). (H) Twenty‐four hours after seeding, a let‐7c‐3p inhibitor and the inhibitor NC were transfected into the cells. After 24 h, anwuligan and TGF‐β were added to the medium, and the cells were incubated at 37°C for another 48 h. The cells were then collected, and a Western blot assay was performed to detect the expression of EMT‐related proteins (*n* = 3). (I) The apoptosis rate of the four groups of cells was detected via annexin V‐FITC/PI staining and flow cytometry (*n* = 3). (J) The morphological changes in the NSCLC cells in the four groups were observed under a light microscope (*n* = 3). (K) A Western blot assay was performed to detect the expression of pro‐apoptosis proteins caspase 3/7/9 and the anti‐apoptotic protein Bcl‐2 in the NSCLC cells of the four groups (*n* = 3).

To investigate whether let‐7c‐3p is necessary for the anti‐tumor effect of anwuligan, 100 nM of a let‐7c‐3p inhibitor (2’‐O‐methyl‐modified), and 100 nM of an inhibitor NC were transfected into A549 and H460 cells before ANW treatment (50 μM); cells in the ANW group were treated with 50 μM ANW in parallel. The cells were divided into four groups (negative control group, ANW group, ANW + let‐7c‐3p inhibitor NC group, and ANW + let‐7c‐3p inhibitor group). The CCK‐8 assay showed that the survival of cells in ANW + let‐7c‐3p inhibitor and negative control groups was obviously higher than that of the cells in ANW and ANW + let‐7c‐3p inhibitor NC groups (Figure 7A). The results of the clone formation assay showed that the number of clones in the ANW + let‐7c‐3p inhibitor and negative control groups was significantly higher than those in the ANW and ANW + let‐7c‐3p inhibitor NC groups (Figure 7B). The results of the EdU assay likewise showed that the proportion of fluorescent cells in the ANW + let‐7c‐3p inhibitor and negative control groups was obviously higher than that of the cells in ANW and ANW + let‐7c‐3p inhibitor NC groups (Figure 7C). The results of the cell cycle distribution analysis showed that the percentages of NSCLC cells in the G0/G1 phase in the ANW + let‐7c‐3p inhibitor and negative control groups were obviously lower than those in the ANW and ANW + let‐7c‐3p inhibitor NC groups (Figure 7D). We previously found that let‐7c‐3p could regulate the PI3K/AKT/mTOR pathway. Here, the western blot results showed that the ANW + let‐7c‐3p inhibitor and negative control groups had notably higher phosphorylation levels of PI3K/AKT/mTOR signaling pathway‐related proteins than the ANW and ANW + let‐7c‐3p inhibitor NC groups (Figure 7E). Thus, inhibiting the expression of let‐7c‐3p could counteract the effect of anwuligan on the survival and proliferation of NSCLC cells and reverse the effect of anwuligan treatment on the phosphorylation of proteins associated with the PI3K/AKT/mTOR signaling pathway.

The results of the wound healing assay indicated that the migration rate of NSCLC cells in the ANW + let‐7c‐3p inhibitor and the negative control groups was obviously higher than that in the ANW + let‐7c‐3p inhibitor NC and ANW groups (Figure 7F). The results of the Transwell assays also revealed that the number of NSCLC cells in the ANW + let‐7c‐3p inhibitor and negative control groups that passed through the filter and Matrigel matrix was notably more than in the ANW+ let‐7c‐3p inhibitor NC and ANW groups (Figure 7G).

TGF‐β (5 ng/mL) was used to treat A549 and H460 cells, and Western blotting was performed to detect the expression levels of EMT‐related marker proteins. The results indicated that the levels of N‐cadherin, MMP3, MMP9, and vimentin in the cells of the ANW + let‐7c‐3p inhibitor and negative control groups were obviously higher than that of the cells in the ANW + let‐7c‐3p inhibitor NC and ANW groups; however, the level of E‐cadherin showed the reverse trend (Figure 7H). In summary, inhibiting the expression of let‐7c‐3p blocked the inhibitory effect of anwuligan on the metastasis and invasion of NSCLC cells.

Annexin V‐FITC/PI staining and flow cytometry were also used to detect the cell apoptosis rates in the four groups. Flow cytometry results showed that the apoptosis rates of A549 and H460 cells in the ANW + let‐7c‐3p inhibitor and negative control groups were remarkably lower than those in the ANW + let‐7c‐3p inhibitor NC and ANW groups (Figure 7I). The morphologies of A549 and H460 cells subjected to the different treatments were observed under a light microscope. The data showed that inhibiting let‐7c‐3p expression blocked the effect of anwuligan treatment on cell atrophy and adhesion (Figure 7J). The four groups of cells were also subjected to western blot analysis to determine the expression of apoptosis‐related proteins. The expression levels of caspase 3/7/9 in the A549 and H460 cells of the control and ANW + let‐7c‐3p inhibitor groups were significantly lower than those in the inhibitor NC and ANW groups. However, the expression levels of Bcl‐2 showed the opposite trend (Figure 7K). In summary, inhibiting let‐7c‐3p expression counteracted the apoptosis‐promoting effect of anwuligan on NSCLC cells.

### 3.8 Nude mouse xenograft tumor model confirmed that anwuligan exerts an anti‐tumor effect by upregulating let‐7c‐3p in vivo

**FIGURE 8:**
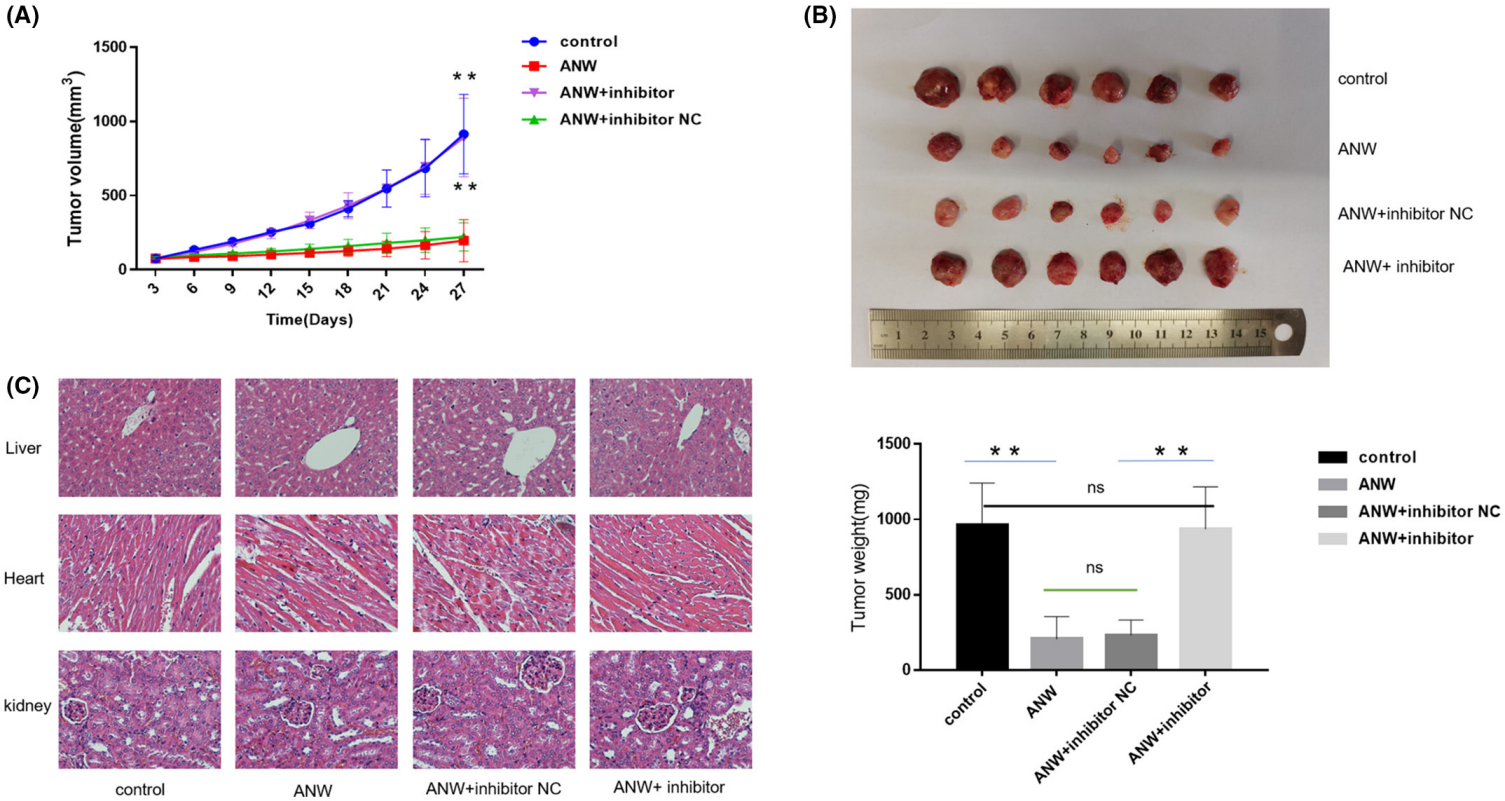
Nude mouse xenograft tumor model confirmed that anwuligan exerts an anti‐tumor effect by upregulating let‐7c‐3p expression in vivo. (A) After intraperitoneal administration of NSCLC cells, the xenograft tumor volume in nude mice was measured every 3 days. (B) After the nude mice were euthanized, the tumors were collected, weighed, and photographed (*n* = 6). (C) The liver, kidney, and heart tissues of the nude mice were sectioned, stained with H&E, and observed under a microscope (*n* = 3).

Previous in vitro experiments have confirmed that ANW exerts an anti‐NSCLC effect by upregulating let‐7c‐3p. Finally, A549 cells were subcutaneously inoculated into BALB/c nude mice to establish a nude mouse xenograft tumor model to test the anti‐tumor effect of ANW in vivo. The mice were divided into four groups (control, ANW, ANW + inhibitor NC, and ANW + let‐7c‐3p inhibitor groups) and each group contained 6 mice. The results showed that the growth rate and weight of the tumors implanted in the ANW and ANW + inhibitor NC groups were significantly lower than those in the control and ANW + inhibitor groups (Figure 8A, B). In addition, H&E staining of the heart, kidney, and liver tissues showed that ANW had no obvious toxicity to the vital organs of nude mice subcutaneously injected with A549 cells (Figure 8C).

## 4 DISCUSSION

In the past decade, multiple studies have shown that natural compounds with biological activity could be promising treatments for human cancers, including lung cancer.^15^ Anwuligan is considered a new P‐glycoprotein drug efflux pump inhibitor, which could improve the bioavailability of paclitaxel. Trace element analysis provided a new understanding of the anti‐tumor effect of the compound.^12^ Therefore, as an active therapeutic drug possessing anti‐cancer potential, further research can clarify the importance of using ANW in complex diseases, such as cancer. Although patients with NSCLC are highly responsive to chemotherapy combined with radiotherapy to improve their long‐term survival, their tumors could potentially be highly resistant to treatment. A few months after diagnosis, the migration and invasiveness of NSCLC cells could lead to metastases in various organs, including the bone, contralateral lung, liver, and brain. NSCLC‐related mortality is usually caused by multiple rapid metastases rather than the primary lung tumor itself.^16^

Toxicity is a well‐known obstacle hindering the potential clinical applications of many natural products. Therefore, this study first verified what concentrations of anwuligan did not induce cytotoxic effects. We found that treatment with less than 100 μM anwuligan had no toxic effect on normal bronchial epithelial cells. However, the viability of A549 and H460 cells treated with anwuligan at this concentration decreased significantly. Treatment with ANW could cause lung cancer cells to stagnate in the G0/G1 phase, inhibiting their proliferation.

The migration and invasiveness of NSCLC cells are the main causes of mortality in patients with NSCLC. Epithelial‐mesenchymal transition (EMT) is the process by which epithelial cells acquire mesenchymal characteristics. EMT is related to tumor occurrence, metastasis, invasion, and resistance to treatment in cancer.^17^ Increased TGF‐β expression in tumor cells can stimulate angiogenesis and promote EMT, thereby increasing the migration and invasion of cancer cells. In addition, TGF‐β can maintain cancer stem cells, and which are associated with tumor progression and poor prognosis.^18^ During EMT, the adhesion and connection proteins in epithelial cells are replaced by N‐cadherin. This protein can provide greater connection flexibility, resulting in cell separation and enhanced cell movement. Studies have shown that cancer cells exhibit “cadherin switch,” indicating the downregulation of E‐cadherin expression and the upregulation of N‐cadherin expression in epithelial cells.^19^

The expression and activity of matrix metalloproteinases (MMPs) were also found to increase in NSCLC tissues compared to normal tissues; as MMPs could regulate the tumor microenvironment. Moreover, MMPs are also associated with tumor metastasis and progression.^20^ MMP9 is one of the most complex MMPs in the gelatinase family. During tissue remodeling, MMP9 can cause the degradation of gelatin and collagen types IV, V, XI, and XVI, which are essential for tumor invasion and metastasis.^21^ The increased expression of MMP3 may also activate other MMPs, such as MMP1 and MMP9, which can promote tumor cell passage through the basement membrane and lead to invasion and metastasis.^20^ Vimentin is an EMT biomarker and intermediate filament protein. Studies have found that vimentin is essential for the early metastasis of lung adenocarcinoma.^22^ In this study, Western blot analysis revealed that anwuligan could reverse EMT and the TGF‐β‐induced changes in the expression of related proteins in A549 and H460 cells, indicating that ANW can inhibit the migration and invasive ability of NSCLC cells.

As one of the best and earliest metal‐based chemotherapeutics, cisplatin can be used to treat multiple solid cancers, including lung cancer.^23^ Cisplatin‐based dual therapy remains the foundation of treatment for most patients with advanced NSCLC.^24^ The incidence of both adverse events and drug resistance has limited the application and effectiveness of cisplatin. Combination therapies have been used to minimize the side effects and drug resistance against cisplatin and have been proven to be more effective against defective cancers.^23^ Our results confirmed that anwuligan treatment could enhance the anti‐tumor effect of cisplatin by increasing cisplatin‐induced apoptosis.

The epigenetic basis of lung cancer is mainly related to changes in miRNA expression. miRNAs are small, single‐stranded, non‐coding RNAs that are important for different cellular processes such as cell development, proliferation, differentiation, growth control, and apoptosis.^25^ Its primary function is to regulate protein translation by binding to a complementary sequence in the 3’‐UTR of its target mRNA, thereby negatively regulating mRNA translation.^26^ miRNAs can efficiently regulate the expression of non‐coding sequences and the expression of other genes involved in the signaling cascade that controls tumorigenesis.^27^ Therefore, miRNAs can be used as potential therapeutic targets. The miRNA let‐7c‐3p is part of the let‐7c family, which has been suggested to have two main biological functions: as essential regulators of terminal differentiation and as tumor suppressors.^28^ Compared with benign breast lesions, let‐7c levels are reduced in breast cancer tissues.^29^ A hospital‐based case–control study involving 120 patients with NSCLC and 360 healthy controls found that the expression of let‐7c in the plasma of patients with NSCLC was downregulated.^30^ Therefore, let‐7c can be used as a tumor biomarker. In this study, after ANW treatment, the expression of several miRNAs changed in NSCLC cells, of which let‐7c‐3p was one of those with the most obvious differences. Both qPCR and preliminary tests revealed that only let‐7c‐3p has a significant role in NSCLC and was affected by drug treatment; hence, we investigated this miRNA further.

The PI3K/AKT/mTOR pathway is an important signaling pathway associated with cell growth, adhesion, and invasion. This pathway is dysregulated in various malignancies, including NSCLC; so, the regulation of this pathway might provide a clue for the treatment of NSCLC.^31^ In this study, bioinformatics analysis revealed that let‐7c‐3p is related to 3‐phosphoinositide biosynthesis. PIK3CA is the catalytic subunit of PI3K, which plays a vital role in maintaining the structure and function of PI3K.^32^ This study confirmed that anwuligan could significantly inhibit the phosphorylation of proteins related to the PI3K/AKT/mTOR signaling pathway, a common pathway targeted by miRNAs. For example, miR‐520a‐3p inhibits the migration, invasion, and proliferation of NSCLC cells through this pathway.^33^ The dual‐luciferase gene reporter system used in this study found that let‐7c‐3p can bind to the 3’‐UTR of PIK3CA; therefore, PIK3CA is a target gene of let‐7c‐3p. We found that let‐7c‐3p regulates the PI3K/AKT/mTOR signaling pathway by inhibiting the expression of PIK3CA, exerting an anti‐tumor effect.

Since we have verified that ANW played an anti‐NSCLC role by upregulating let‐7c‐3p expression, we also explored the specific mechanism by which downregulating let‐7c‐3p could counteract this effect. The results indicated that inhibiting the expression of let‐7c‐3p could reverse the ANW‐induced phosphorylation PI3K/AKT/mTOR signaling pathway‐related proteins, reduce the level of apoptosis‐related protein caspase3/7/9, and promote the expression of EMT‐related proteins. Therefore, the downregulation of let‐7c‐3p can offset the anti‐NSCLC effect of ANW by regulating the protein expression of downstream signaling pathways. These data further demonstrate that ANW exerts anti‐tumor effects by upregulating let‐7c‐3p expression. Finally, we confirmed the anti‐NSCLC effect of ANW through in vivo experiments. The results further confirmed that let‐7c‐3p is a target of ANW in NSCLC, and that ANW treatment did not cause obvious side effects in nude mice. Further animal studies will provide a preliminary basis for the safety of ANW treatment.

In conclusion, through in vivo and in vitro experiments, this study confirmed the important role of ANW in inhibiting NSCLC through let‐7c‐3p. It also provided possible targets and new treatment ideas for the treatment of NSCLC, which are worthy of further study.

## 5 CONCLUSION

Anwuligan inhibited the growth and metastasis of NSCLC cells in vivo and in vitro by upregulating let‐7c‐3p expression through the PI3K/AKT/mTOR signaling pathway. Moreover, PIK3CA is the main target gene of let‐7c‐3p.

## AUTHOR CONTRIBUTIONS

**Huikun niu:** Conceptualization (equal); data curation (equal); investigation (equal); methodology (equal); writing – original draft (equal); writing – review and editing (equal). **Dexiang Wang:** Conceptualization (equal); data curation (equal); formal analysis (equal); investigation (equal); methodology (equal); writing – original draft (equal); writing – review and editing (equal). **tingting wen:** Conceptualization (equal); data curation (equal); formal analysis (equal); methodology (equal); project administration (equal). **Han Liu:** Data curation (equal); methodology (equal). **Jing Jie:** Conceptualization (equal); data curation (equal); methodology (equal). **Lei Song:** Conceptualization (equal); data curation (equal); formal analysis (equal); investigation (equal); methodology (equal). **Dan Li:** Conceptualization (equal); formal analysis (equal); investigation (equal); methodology (equal); project administration (equal).

## ETHICS APPROVAL

All animal experiments were approved by the Animal Protection and Utilization Committee of the First Hospital of the Jilin University.

## Data Availability

All data generated or analyzed during this study are included in this published article.

## References

[1] Sung H , Ferlay J , Siegel RL , et al. Global Cancer Statistics 2020: GLOBOCAN estimates of incidence and mortality worldwide for 36 cancers in 185 countries. CA Cancer J Clin. 2021;71:209‐249. 33538338 10.3322/caac.21660

[2] Lu TX , Rothenberg ME . MicroRNA. J Allergy Clin Immunol. 2018;141:1202‐1207. 29074454 10.1016/j.jaci.2017.08.034 PMC5889965

[3] Chen L , Heikkinen L , Wang C , Yang Y , Sun H , Wong G . Trends in the development of miRNA bioinformatics tools. Brief Bioinform. 2019;20:1836‐1852. 29982332 10.1093/bib/bby054 PMC7414524

[4] Ganju A , Khan S , Hafeez BB , et al. MiRNA nanotheraprutics for cancer. Drug Discovery Today. 2017;22(2):424‐432. 27815139 10.1016/j.drudis.2016.10.014 PMC5309208

[5] Rupaimoole R , Slack FJ . MicroRNA therapeutics: towards a new era for the management of cancer and other diseases. Nat Rev Drug Discov. 2016;16(3):203‐222. 10.1038/nrd.2016.246 28209991

[6] Iqbal MA , Arora S , Prakasam G , Calin GA , Syed MA . MicroRNA in lung cancer: role, mechanisms, pathways and therapeutic relevance. Mol Aspects Med. 2019;70:3‐20. 30102929 10.1016/j.mam.2018.07.003

[7] Wu KL , Tsai YM , Lien CT , Kuo PL , Hung AJ . The roles of MicroRNA in lung cancer. Int J Mol Sci. 2019;7(20):1611. 10.3390/ijms20071611 PMC6480472 30935143

[8] Xu F , Na L , Li Y , Chen L . Roles of the PI3K/AKT/mTOR signalling pathways in neurodegenerative diseases and tumours. Cell Biosci. 2020;10:54. 32266056 10.1186/s13578-020-00416-0 PMC7110906

[9] Tewari D , Patni P , Bishayee A , Sah AN , Bishayee A . Natural products targeting the PI3K‐Akt‐mTOR signaling pathway in cancer: a novel therapeutic strategy. Semin Cancer Biol. 2019;80:1‐17. 31866476 10.1016/j.semcancer.2019.12.008

[10] Tan AC . Targeting the PI3K/Akt/mTOR pathway in non‐small cell lung cancer (NSCLC). Thorac Cancer. 2020;11:511‐518. 31989769 10.1111/1759-7714.13328 PMC7049515

[11] Sun LR , Zhou W , Zhang HM , et al. Modulation of multiple signaling pathways of the plant‐derived natural products in cancer. Front Oncol. 2019;9:1153. 31781485 10.3389/fonc.2019.01153 PMC6856297

[12] Paul S , Hwang JK , Kim HY , Jeon WK , Chung C , Han JS . Multiple biological properties of macelignan and its pharmacological implications. Arch Pharm Res. 2013;36:264‐272. 23435944 10.1007/s12272-013-0048-z

[13] Han YS , Kim MS , Hwang JK . Macelignan inhibits histamine release and inflammatory mediator production in activated rat basophilic leukemia mast cells. Inflammation. 2012;5(35):1723‐1731. 10.1007/s10753-012-9490-1 22729280

[14] Song Y , Zhang Y , Duan XY , et al. Pharmacokinetics and tissue distribution of anwuligan in rats after intravenous and intragastric administration by liquid chromatography‐mass spectrometry. Molecules. 2019;1(25):39. 10.3390/molecules25010039 PMC6983174 31861927

[15] Yan Y , Su W , Zeng S , et al. Effect and mechanism of tanshinone I on the radiosensitivity of lung cancer cells. Mol Pharm. 2018;15:4843‐4853. 30216081 10.1021/acs.molpharmaceut.8b00489

[16] Webb JD , Simon MC . Novel insights into the molecular origins and treatment of lung cancer. Cell cycle. 2010;9:4098‐4105. 20962595 10.4161/cc.9.20.13588 PMC3055194

[17] Ievgenia P , Cédric B . EMT transition states during tumor progression and metastasis. Trends Cell Biol. 2018;29:212‐226. 30594349 10.1016/j.tcb.2018.12.001

[18] Morikawa M , Derynck R , Miyazono K . TGF‐β and the TGF‐β family: context‐dependent roles in cell and tissue physiology. Cold Spring Harb Perspect Biol. 2016;8:a021873. 27141051 10.1101/cshperspect.a021873 PMC4852809

[19] Wheelock MJ , Johnson KR . Cadherins as modulators of cellular phenotype. Ann Rev Cell Dev Biol. 2003;19:207‐235. 14570569 10.1146/annurev.cellbio.19.011102.111135

[20] Fang S , Xia J , Rui W , et al. Polymorphisms in the MMP1 and MMP3 promoter and non‐small cell lung carcinoma in North China. Carcinogenesis. 2005;26(2):481‐486. 15528217 10.1093/carcin/bgh327

[21] Mondal S , Adhikari N , Banerjee S , Amin SA , Jha T . Matrix metalloproteinase‐9 (MMP‐9) and its inhibitors in cancer: a minireview. Eur J Med Chem. 2020;194:112260. 32224379 10.1016/j.ejmech.2020.112260

[22] Richardson AM , Havel LS , Koyen AE , et al. Vimentin is required for lung adenocarcinoma metastasis via heterotypic tumor cell‐cancer associated fibroblast interactions during collective invasion. Clin Cancer Res. 2018;24:420‐432. 29208669 10.1158/1078-0432.CCR-17-1776 PMC5771825

[23] Ghosh S . Cisplatin: the first metal based anticancer drug. Bioorg Chem. 2019;88:102925. 31003078 10.1016/j.bioorg.2019.102925

[24] Fennell DA , Summers Y , Cadranel J , et al. Cisplatin in the modern era: the backbone of first‐line chemotherapy for non‐small cell lung cancer. Cancer Treat Rev. 2016;44:42‐50. 26866673 10.1016/j.ctrv.2016.01.003

[25] Bersimbaev R , Pulliero A , Bulgakova O , Asia K , Izzotti A . Radon biomonitoring and microRNA in lung cancer. Int J Mol Sci. 2020;21:2154. 32245099 10.3390/ijms21062154 PMC7139524

[26] Hayes J , Peruzzi PP , Lawler S . MicroRNAs in cancer: biomarkers, functions and therapy. Trends Mol Med. 2014;20:460‐469. 25027972 10.1016/j.molmed.2014.06.005

[27] Ling H , Fa Bbri M , Calin GA . MicroRNAs and other non‐coding RNAs as targets for anticancer drug development. Nat Rev Drug Discov. 2013;12:847‐865. 24172333 10.1038/nrd4140 PMC4548803

[28] Lee H , Han S , Chang SK , Lee D . Biogenesis and regulation of the let‐7 miRNAs and their functional implications. Protein Cell. 2016;7:100‐113. 26399619 10.1007/s13238-015-0212-y PMC4742387

[29] Spolverini A , Fuchs G , Bublik DR , Oren M . let‐7b and let‐7c microRNAs promote histone H2B ubiquitylation and inhibit cell migration by targeting multiple components of the H2B deubiquitylation machinery. Oncogene. 2017;36:5819‐5828. 28604753 10.1038/onc.2017.187 PMC5600258

[30] Dou H , Yan W , Gang S , Song Z . Decreased plasma let‐7c and miR‐152 as noninvasive biomarker for non‐small‐cell lung cancer. Int J Clin Exp Med. 2015;8:9291‐9298. 26309587 PMC4538081

[31] Salehi M , Movahedpour A , Tayarani A , Shabaninejad Z , Mirzaei H . Therapeutic potentials of curcumin in the treatment of non‐small‐cell lung carcinoma. Phytother Res. 2020;34:2557‐2576. 32307773 10.1002/ptr.6704

[32] Alex MM , Enriqueta F . PI3K pathway in NSCLC. Front Oncol. 2011;1:55. 22655251 10.3389/fonc.2011.00055 PMC3356073

[33] Lv X , Li CY , Han P , Xu XY . MicroRNA‐520a‐3p inhibits cell growth and metastasis of non‐small cell lung cancer through PI3K/AKT/mTOR signaling pathway. Eur Rev Med Pharmacol Sci. 2018;22(8):2321‐2327. 29762835 10.26355/eurrev_201804_14822

